# Our experience of lung resection in patients who decline blood transfusion for religious reasons

**DOI:** 10.1007/s11748-021-01589-2

**Published:** 2021-02-06

**Authors:** Hironori Takagi, Satoshi Muto, Hikaru Yamaguchi, Hayato Mine, Yuki Ozaki, Naoyuki Okabe, Yuki Matsumura, Yutaka Shio, Hiroyuki Suzuki

**Affiliations:** Department of Chest Surgery, Fukushima Medical University School of Medicine, 1 Hikarigaoka, Fukushima, 960-1295 Japan

**Keywords:** Jehovah’s witness, Lung resection, Lung cancer, Autologous blood transfusion, *EGFR* mutation

## Abstract

**Objective:**

Surgical treatment for patients who refuse blood transfusion due to religious beliefs is an important issue related to medical safety. Few reports have examined pulmonary surgery for these patients, and we analyzed clinical characteristics in such cases.

**Methods:**

Ten Jehovah’s Witness (JW) patients with lung tumor resection who declined blood transfusion for religious reasons between December 2013 and February 2020 at the Fukushima Medical University Hospital were included. Median total intraoperative blood loss was 17.5 mL (range 5–150 mL). Fibrin glue was used intraoperatively for 8 patients. Final pathological examination revealed pulmonary adenocarcinoma in 9 cases and metastasis of bladder cancer in 1 case. In 8 patients with pulmonary adenocarcinoma examined for epidermal growth factor receptor (*EGFR*) gene mutation, 6 cases showed mutation. No patients had serious complications, but 1 patient displayed temporary anemia due to postoperative hemorrhagic gastrointestinal ulcer.

**Result and conclusions:**

Our findings confirm that pulmonary resection is feasible and safe for JW patients if performed by experienced medical staff. However, awareness of complications associated with perioperative bleeding is important. Each JW patient should be interviewed individually and every available perioperative option aimed at blood-sparing management, including use of blood coagulation factors and fibrinogen concentrates, should be carefully discussed and clarified. In this study, the *EGFR* gene mutation rate was higher than usual for cases of lung adenocarcinoma. Further studies are necessary to assess clinical features in JW patients with lung cancer.

## Introduction

Given the risks of perioperative bleeding and the refusal of blood transfusion on religious grounds, surgery for Jehovah’s Witness (JW) patients always represents a clinical challenge and has generated various ethical and legal concerns. Some reports have described perioperative prevention of bleeding and anemia for JW patients, but indicators of strategies for malignant tumor surgery in JW patients have remained lacking. Some recent reports have described experiences with JW, such as case reports of esophageal cancer surgery [1] and sigmoid colon cancer [2]. However, very few reports have examined issues with pulmonary surgery. The aim of this study was to investigate the clinical issues specific to pulmonary surgery and cancer treatment for JW patients. This was a retrospective review of medical records for 10 JW patients who underwent pulmonary resection at our institution.

## Materials and methods

We enrolled 10 JW patients who underwent pulmonary resection for lung tumor between December 2013 and February 2020 at Fukushima Medical University Hospital.

We explained the non-transfusion and exemption from responsibility, and obtained consent for surgery preoperatively using documents based on the guidelines for religious rejection of blood transfusion in Japan [3], as approved by the ethics committee of this hospital. In addition, we confirmed the details of those blood products that each individual patient would permit.

The surgical procedure was determined according to National Comprehensive Cancer Network guidelines [4]. No cases were encountered in which the surgical procedure was changed because of possible surgical invasiveness or bleeding risk.

Preoperatively, mean ± SD hemoglobin level was 13.25 ± 1.04 g/dL and no patient had optimization of hemoglobin, such as treatment with iron.

## Results

This study included 10 patients (2 men, 8 women) (Table 1). Median age was 70 years (range 51–82 years), and 3 patients had undergone previous cancer surgery (rectal cancer resection, breast cancer resection, and bladder cancer transurethral resection: 1 patient each). Two patients had diabetes and 1 patient had liver cirrhosis or antineutrophil cytoplasmic antibody-related vasculitis. The approach was lobectomy in 7 patients (3 patients with open surgery, 2 patients with video-assisted thoracic surgery (VATS), and 2 patients with robot-assisted thoracic surgery) and wedge resection in 3 patients (1 patient with open surgery, 2 patients with VATS). Median blood loss was 17.5 mL (range 5–150 mL). In each case, the specific blood products rejected by the individual were different.

**Table 1:** Patient characteristics

Case	Age (years)	Sex	Smoking (BI)	Preoperative Hb (g/dL)	c-Stage (TNM 7th)	Past history	Procedures	Operating time (min)	Bleeding (ml)	p-Stage (TNM 7th)	EGFR gene mutation	Complications (Clavien-Dindo)	Outcome (postop. months)
1	51	F	0	12.9	c-T1aN0M0 stage IA	Appendectomy	VATS-RLL	174	55	p-T1bN0M0 stage IB lung adenocarcinoma	Del 19	Air leakage (G2)	Recurrence (4 mo.) alive (76 mo.)
2	70	F	0	14.3	c-T1aN0M0 stage IA	Rectal cancer resection	VATS-RUL	148	5	p-T1aN0M0 stage IA lung adenocarcinoma	NA		No recurrence (48 mo.) alive (48 mo.)
3	70	F	0	12.2	c-T2aN1M0 stage IIA	TB endometriosis	RUL	179	35	p-T3N1M0 stage IIIA lung adenocarcinoma	ND		Recurrence (2 mo.) alive (27 mo.)
4	71	F	0	14.3	c-T1bN0M0 stage IB	Breast cancer resection	RLL	148	25	p-T1bN0M0 stage IB lung adenocarcinoma	L858R		No recurrence (27 mo.) alive (27 mo.)
5	76	F	200	14.5	c-T1bN0M0 stage IB	Uterine myoma resection	LUL	175	35	p-T1aN0M0 stage IA lung adenocarcinoma	L858R	hyponatremia (G2)	No recurrence (23 mo.) alive (23 mo.)
6	82	M	500	11.1	c-T1aN0M0 stage IA	DM AMI	Open-partial	246	150	p-T3N1M0 stage IIIA lung adenocarcinoma	L861Q	gastrointestinal ulcer (G2)	recurrence (2 mo.) dead (6 mo.)
7	69	F	0	13.2	c-T1aN0M0 stage IA	Adrenal tumor (non-functional)	RATS-RUL	179	10	p-T1aN0M0 stage IA lung adenocarcinoma	L858R		No recurrence (9 mo.) alive (9 mo.)
8	75	F	0	13.7	c-T1aN0M0 stage IA	Appendectomy	RATS-RUL	168	10	p-T1aN0M0 stage IA lung adenocarcinoma	ND		No recurrence (8 mo.) alive (8 mo.)
9	65	M	300	13.3	Metastasis (bladder cancer)	DM	VATS-partial	85	10	Metastasis (bladder cancer)	–		NA
10	79	F	0	13.0	c-T1aN0M0 stage IA	HCC (chemo.) NASH	VATS-partial	90	10	p-T1aN0M0 stage IA lung adenocarcinoma	Del 19		No recurrence (3 mo.) alive (3 mo.)

*BI* Brinkman index, *Hb* hemoglobin, *TB* tuberculosis, *DM* diabetes mellitus, *AM*I acute myocardial infarction, *HCC* hepatocellular carcinoma, *NASH* non-alcoholic steatohepatitis, *VATS* video-assisted thoracic surgery, *RATS* robot-assisted thoracic surgery, *RUL* right upper lobectomy, *RLL* right lower lobectomy, *LUL* left upper lobectomy, ND not detected, *NA* not applicable

In the preoperative confirmation process, 5 patients indicated they would not accept autologous blood collected from the surgical field using an instrument (cell saver) that is physically contiguous with the body and 3 patients stated they would accept autotransfusion of preoperatively stored blood (Table 2). No patients were prepared for autologous blood transfusion or used cell saver due to the expected surgical procedure and invasiveness of the operation. No patient received blood transfusion, but fibrinogen products were used for 8 patients to prevent air leakage. Pathologic examination revealed 9 cases of pulmonary adenocarcinoma and 1 case of metastatic bladder cancer. Eight of the 9 patients with lung cancer were screened for epidermal growth factor receptor (EGFR) gene mutation, with 6 patients (75%) showing positive results for *EGFR* mutation.

**Table 2:** Permission for blood products

Blood transfusion method or blood product (*n* = 11)	Preoperatively stored autologous	Intraoperatively collected autologous	Fibrinogen products
Number of patients with permission	3	5	9
Number of applications	0	0	8

No patients showed serious complications, but 1 patient experienced transient anemia (Clavien–Dindo classification grade 2; hemoglobin, 7.4 g/dL) due to bleeding from a gastrointestinal ulcer after taking non-steroidal anti-inflammatory drugs for postoperative pain. This case improved with conservative treatment using a proton pump inhibitor and iron infusion.

Since 9 of the 10 patients were refused surgery at another hospital due to their refusal of blood transfusion and were referred to our hospital, they are being followed-up postoperatively at the referring institutions. Because of concerns about anemia as an adverse event, only 1 patient received adjuvant chemotherapy.

After a median follow-up of 23 months (range 3–76 months) from resection in 9 patients with lung cancer, 3 patients experienced recurrence (p-stage IIIA, 2 patients; p-stage IB, 1 patient). All these recurrences occurred within the first year after operation and 1 of these patients died of lung cancer 6 months postoperatively. As of the time of writing, 5 patients with p-stage IA and 1 patient with p-stage IB remain alive without recurrence.

## Discussion

There are approximately 8.5 million JWs all over the world, with a community of about 210,000 members in Japan [5]. The number of followers is increasing globally, but is gradually decreasing in Japan. JWs refuse blood product transfusions based on their religious beliefs. In some clinical situations such as operations, this can pose both healthcare and ethical challenges.

In Japan, 1 patient who underwent hepatectomy was given a transfusion to avoid the risk of anemia, even though he had declined transfusion preoperatively. In February 2000, the Supreme Court ruled that the individual right to self-determination was more important than the risk to his life [6]. This ruling received social attention. Guidelines were, therefore, established for patients with religious refusal of blood transfusion in 2008 [3]. However, detailed treatment plans have been entrusted to each institution, and invasive treatment for JWs remains a medical and ethical issue.

Some reports have described surgical treatment for JWs [1, 2, 7–9]. Although case reports have described abdominal surgery, cardiovascular surgery, and liver or lung transplantation, very few have mentioned pulmonary surgery, especially cases of lung cancer [10, 11].

One problem in the surgical treatment of JW patients is the issue of treatment plans accounting for bleeding risk, anemia, and surgical invasion. As shown in this study, although early-stage lung cancer can be safely resected as in normal operations, treatment plans for surgery for locally advanced lung cancer are difficult to determine because of the invasiveness. Extracorporeal circulation with autologous blood transfusion using cell savers, which are used in cardiovascular surgery cases at risk of massive bleeding, has been thought to be associated with dissemination or recurrence [12]. Therefore, operations on JW patients with lung cancer place great stress on the operator.

**Fig. 1:**
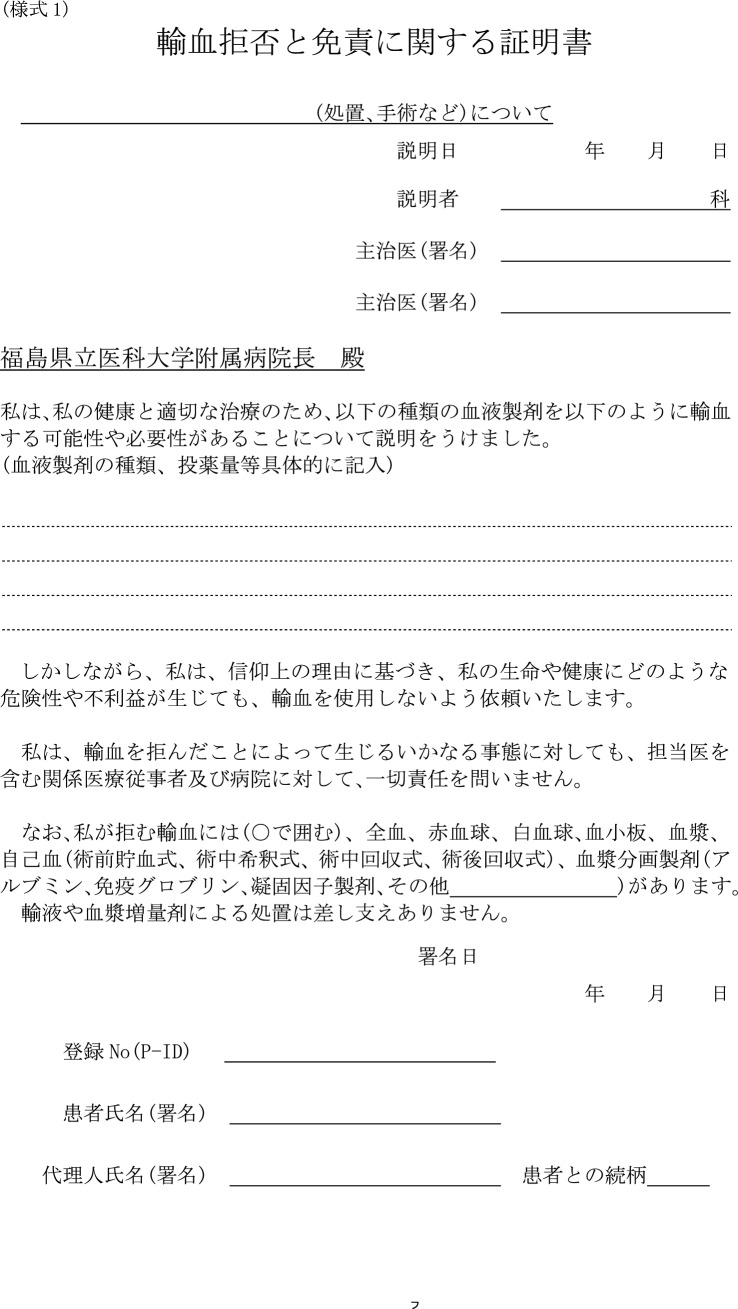
Disclaimer documents from our institution. In our hospital, we obtain the consent of each JW patient to undergo surgery under a policy of absolute bloodlessness using this disclaimer document based on the guidelines for religious rejection of blood transfusion in Japan before the operation. We also check with each JW patient one by one for the types of blood products they reject, including coagulation factors

The following measures could be considered for massive bleeding and severe anemia in lung cancer operations, in reference to the reports of resection for other malignant tumors. One is autologous blood transfusion through closed extracorporeal circulation. Depending on the beliefs of the individual, JW patients can often undergo salvage autologous blood transfusions that are not withdrawn from the body circulation. According to recent reports on salvage autologous blood transfusions with several cancers, there is no conclusive evidence that this process can induce metastases or dissemination [13–15]. Massive bleeding can directly lead to fatality in cases where blood transfusion is refused. Autologous blood transfusions are thus an option as a countermeasure to the risk of bleeding. The details of precisely which autologous blood transfusions and blood products the patient will find acceptable must be confirmed before the operation. In our hospital, we respect the wishes and obtain the express consent of the JW patient to undergo operations under a policy of absolute bloodlessness even in life-threatening situations, using disclaimer documents based on the guidelines for religious rejection of blood transfusion in Japan before the operation (Fig. 1). We specifically explain to JW patients the surgical procedures, the expected intra- and postoperative complications related to bleeding, and the possibility of complications in the event of non-transfusion. In addition, we have the JW patient confirm that they will not place any responsibility on the medical staff or our hospital, even if a life-threatening condition results from their decision to decline blood transfusions. We also check with each JW patient individually regarding the types of blood products they reject, including coagulation factors.

The other measure is perioperative blood management to avoid fatal anemia. In particular, in cases of preoperative anemia, vitamins (B6, B12, folic acid) and iron are administered to avoid intra- and postoperative anemia. This method has already been actively incorporated in abdominal surgery and its usefulness has been reported [7, 16–18]. The utility of erythropoietin administration has been reported [17, 18], but problems have been encountered with coverage by medical insurance in Japan.

Induction chemotherapy for lung cancer could be considered as an additional supplement. In recent years, cases have been described involving locally advanced colorectal cancer with religious refusal of blood transfusion that have become resectable by induction chemotherapy [2]. Even in cases of lung cancer, induction chemotherapy may be considered in cases of surgery for locally advanced non-small cell lung cancer, but few reports have clarified its effectiveness [19, 20]. In the adverse events from induction chemotherapy, careful attention should be paid to myelosuppression, and anemia in particular, among cases of transfusion rejection. Recently, some surgical reports and clinical trials have described the benefits of induction therapy with immune checkpoint inhibitors in lung cancer [21–23]. In the future, this may represent an effective option for tumor down-staging and avoiding extensive surgery.

By preoperatively considering alternatives to blood transfusion, we were able to perform appropriate surgeries while preserving the rights of patients who refuse blood transfusion. Such considerations could also contribute to reducing the stress on the operator. In addition, non-transfusion surgery is beneficial not only for cases of religious refusal of blood transfusion, but also for all surgical cases, due to the risk of infection and immunological side effects [24].

In this study, the rate of *EGFR* mutation-positive findings in cases of lung adenocarcinoma was 75%, higher than in the general population [25, 26]. No case studies of religious refusal of blood transfusion in lung cancer have been reported, and patient backgrounds have been unclear. Our results suggest that this is due to the large proportion of non-smoking women among JW patients. Tissue biopsy, screening for *EGFR* mutations, and administration of EGFR tyrosine kinase inhibitor, rather than radical resection, may improve the prognosis for JW patients at higher risk of bleeding. However, this was a retrospective analysis of a small number of cases, and analysis of a large number of cases and patient backgrounds, including gene mutations, is desirable.

## References

[1] Inoue S, Miyoshi T, Aoyama M, Hino N, Yamasaki S (2015). A case of thoracic esophageal cancer undergone esophagectomy after induction chemotherapy in a Jehovah’s Witness. J Med Invest.

[2] Motegi S, Kawahara M, Tonoike Y, Kitami C, Makino S, Nishimura A (2018). A case of radical resection after CapeOX therapy for locally advanced sigmoid colon cancer with anemia and abscess formation in a Jehovah’s witness. Gan To Kagaku Ryoho.

[3] Japan Surgical Society (2008). Guidelines on religious blood transfusion refusal http://www.jssoc.or.jp/other/info/info20080523-1.pdf. Accessed 17 Aug 2020

[4] National Comprehensive Cancer Network (NCCN) (2018) NCCN clinical practice guidelines in oncology (NCCN Guidelines^®^) Non-small cell lung cancer https://www2.tri-kobe.org/nccn/guideline/lung/english/non_small.pdf. Accessed 17 Aug 2020

[5] JW. ORG Jehovah's Witnesses (2019) 2019 Grand Totals https://www.jw.org/en/library/books/2019-service-year-report/2019-grand-totals/ Accessed 17 Aug 2020

[6] JW. ORG Jehovah’s Witnesses watchtower ONLINE LIBRARY (2000) https://wol.jw.org/ja/wol/d/r7/lp-j/102000687#h=8:0-9:499 Accessed 17 Aug 2020

[7] Costanzo D, Bindi M, Ghinolfi D, Esposito M, Corradi F, Forfori F (2020). Liver transplantation in Jehovah’s witnesses: 13 consecutive cases at a single institution. BMC Anesthesiol.

[8] Valle FH, Pivatto Júnior F, Gomes BS, Freitas TM, Giaretta V, Gus M (2017). Cardiac surgery in Jehovah’s witness patients: experience of a Brazilian tertiary hospital. Braz J Cardiovasc Surg.

[9] Moulton LJ, Rose PG, Mahdi H (2017). Adverse post-operative outcomes in Jehovah’s witnesses with gynecologic cancer within 30 days of surgery: a single institution review of 36 cases. Gynecol Oncol Rep.

[10] Cerezo Madueño F, Arango Tomás E, Salvatierra VÁ (2013). Lung transplant in Jehovah’s witness patient. J Thorac Cardiovasc Surg.

[11] Rispoli M, Bergaminelli C, Nespoli MR, Esposito M, Mattiacci DM, Corcione A (2016). Major thoracic surgery in Jehovah’s witness: a multidisciplinary approach case report. Int J Surg Case Rep.

[12] Blajchman MA (1998). Immunomodulatory effects of allogeneic blood transfusions: clinical manifestations and mechanisms. Vox Sang.

[13] Kumar N, Chen Y, Zaw AS, Nayak D, Ahmed Q, Soong R (2014). Use of intraoperative cell-salvage for autologous blood transfusions in metastatic spine tumour surgery: a systematic review. Lancet Oncol.

[14] Raval JS, Nelson JB, Woldemichael E, Triulzi DJ (2012). Intraoperative cell salvage in radical prostatectomy does not appear to increase long-term biochemical recurrence, metastases, or mortality. Transfusion (Paris).

[15] Wu WW, Zhang WY, Zhang WH, Yang L, Deng XQ, Ou MC (2019). Survival analysis of intraoperative blood salvage for patients with malignancy disease: a PRISMA-compliant systematic review and meta-analysis. Medicine (Baltimore).

[16] Goodnough LT, Maniatis A, Earnshaw P, Benoni G, Beris P, Bisbe E (2011). Detection, evaluation, and management of preoperative anaemia in the elective orthopaedic surgical patient: NATA guidelines. Br J Anaesth.

[17] Scharman CD, Burger D, Shatzel JJ, Kim E, DeLoughery TG (2017). Treatment of individuals who cannot receive blood products for religious or other reasons. Am J Hematol.

[18] Chaturvedi S, Koo M, Dackiw L, Koo G, Frank SM, Resar LMS (2019). Preoperative treatment of anemia and outcomes in surgical Jehovah’s witness patients. Am J Hematol.

[19] Scagliotti GV, Pastorino U, Vansteenkiste JF, Spaggiari L, Facciolo F, Orlowski TM (2012). Randomized phase III study of surgery alone or surgery plus preoperative cisplatin and gemcitabine in stages IB to IIIA non-small-cell lung cancer. J Clin Oncol.

[20] Rendina EA, Venuta F, De Giacomo T, Ciccone AM, Ruvolo G, Coloni GF (1999). Induction chemotherapy for T4 centrally located non-small cell lung cancer. J Thorac Cardiovasc Surg.

[21] Kawai H, Saito Y, Demura R, Odaka H, Takahashi S, Takahashi K (2018). Case of advanced pulmonary squamous cell carcinoma cured by resection through preoperative induction of immune checkpoint inhibitor. Thoracic Cancer.

[22] Forde PM, Chaft JE, Smith KN, Anagnostou V, Cottrell TR, Hellmann MD (2018). Neoadjuvant PD-1 blockade in resectable lung cancer. N Engl J Med.

[23] Ren S, Wang C, Shen J, Zhu C (2020). Neoadjuvant immunotherapy with resectable non-small cell lung cancer: recent advances and future challenges. J Thorac Dis.

[24] Shander A, Javidroozi M, Perelman S, Puzio T, Lobel G (2012). From bloodless surgery to patient blood management. Mt Sinai J Med.

[25] Midha A, Dearden S, McCormack R (2015). EGFR mutation incidence in non-small-cell lung cancer of adenocarcinoma histology: a systematic review and global map by ethnicity (mutMapII). Am J Cancer Res.

[26] Dearden S, Stevens J, Wu YL, Blowers D (2013). Mutation incidence and coincidence in non small-cell lung cancer: meta-analyses by ethnicity and histology (mutMap). Ann Oncol.

